# Editorial: Green Perspective of Nano-Biotechnology: Nanotoxicity Horizon to Biomedical Applications

**DOI:** 10.3389/fbioe.2022.919226

**Published:** 2022-05-30

**Authors:** Suresh K. Verma, Mrutyunjay Suar, Yogendra Kumar Mishra

**Affiliations:** ^1^ Condensed Matter Theory Group, Materials Theory Division, Department of Physics and Astronomy, Uppsala University, Uppsala, Sweden; ^2^ KIIT School of Biotechnology, KIIT University, Bhubaneswar, India; ^3^ Mads Clausen Institute, NanoSYD, University of Southern Denmark, Sønderborg, Denmark

**Keywords:** green chemistry, nanotoxicity, nano-biotechnology, nanoparticles, biocompatibility

The revelations of green and sustainable approaches in the field of nanotechnology and biotechnology have been proved to be beneficial tools for innovations in and the implementation of new technologies. Green nanotechnology techniques have progressed to the synthesis and production of many organic and inorganic nanoparticles and nanocomposites. Moreover, the techniques have opened the door for the utilization of renewable and natural environmental resources for designing and deploying new nanomaterials-based products. Natural compounds like cellulose and plant biomolecules have been recognized as sourcing agents for the synthesis and designing of nanomaterials. The designed nanomaterials from green synthesis have been investigated for their deployment in biomedical and environmental applications. However, it is important to investigate and explore the bio-compatibility and eco-compatibility aspects of the designed nanomaterials using different *in vitro* and *in vivo* scientific models. This research and review article Research Topic focuses on high-quality research emphasizing the green perspective of Nano-biotechnology expanding their horizon to the utilization of biocompatible natural biomolecules for different biomedical and environmental applications.

Green synthesis of inorganic nanoparticles especially metal oxide nanoparticles was one of the focused works in the Research Topic. Berehu et al. synthesized ZnO nanoparticles using the leaf extract of *Swertia chirayita* with proper characterization through different techniques and formed ZnO nanoparticles in a different solvent medium. They also proved the biomedical properties of ZnO nanoparticles as an anti-cancerous agent in colon cells and kidney cells. The mechanistic investigation proved that the anti-cancerous activities of ZnO nanoparticles were carried by the process of apoptosis with a significantly elicited expression of E-cadherin and reduced expression of vimentin and CDK-1. The findings advanced knowledge of green synthesis of ZnO nanoparticles using a herb with anti-cancerous properties. The environmental application of the ZnO nanoparticles along with selenium nanoparticles was studied by Fasil et al. explaining the utility of nanoparticles as dietary micronutrients for aquatic animal growth in the Zebrafish model. They explained that the modularly synergistic effect of ZnO NP and SeNP in the basal feed of zebrafish can improve the growth performance and development of zebrafish. The nanoparticles synthesized using the high-energy ball milling method were fed to zebrafish and found to elicit growth-related gene expression with a reduced level of reactive oxygen species production. The study explains the environmental and eco-compatible uses of green synthesized nanoparticles.

Exploration of new phytometabolites from natural plant resources for biomedical and environmental applications is an important aspect of green and sustainable technologies. Kaushik et al. explored the potential biocompatibility of ethanolic extract of plants named *Hibiscus syriacus* and *Cinnamomum loureirii* Nees. They investigated the potential use of these extracts for the synthesis of nanomaterials and explained their cytotoxicity in human skin cells with mechanistic details. The investigation revealed the presence of polyphenols and flavonoids along with vanilloloside epicatechin in the *Cinnamomum* Nees extracts, which were capable of inducing cell death via the process of apoptosis through cell cycle arrest and nitric oxide production. The study showed their potential as an anti-cancerous agent.

Extending the utility of the different metabolites produced by natural resources, Mishra et al. reviewed the synthesis, production, and application of cellulose for biomedical and environmental purposes. Cellulose is one of the important constituents of all the plants, bacteria, and microbes, which can be implicated in different applications. The review focused on the bacterial cellulose and defined in detail the synthesis of cellulose form bacterial sources such as Rhizobium, *Azotobacter*, Agrobacterium, *Salmonella*, Aerobacter, *Acetobacter*, Achromobacter, and *Escherichia* sp. Moreover, along with the focus on non-bacterial cellulose, they explained the molecular mechanism behind the synthesis of cellulose and explored the potential use of mechanistic knowledge for cellulose extraction from organic waste for food, biomedical and environmental applications. The knowledge was further extended for nanocellulose as an application biomedical scaffold by Mishra et al. The review article assessed the technological characteristics (chemistry of cellulose, nanocellulose producing methods, its purity, and biological aspects including toxicity in drug formulation). The definition of nanocellulose can be determined as a nano form of cellulose crystals that can be modified for different biomedical and environmental applications. The review presented by Mishra et al. enlightened the use and production of nanocellulose with legal aspects in REACH (Registration, Evaluation, Authorization, and Restriction of Chemicals) regulation by the European Union, EMA (European Medicine Agency). They defined the chemistry, synthesis, and biological properties of nanocellulose for different applications of biomedicine like drug delivery and medical products. The explanation was extended to views of the use of cellulose and nanocellulose within the regulation of European medical agencies for better biomedical and environmental usage.

In summary, this Research Topic covers recent advances in the field of nanobiotechnology along with new insights into the exploration and dissemination of phytometabolites for nanomaterials synthesis and production for different biomedical and environmental applications. The editors aim for this Research Topic to provide a “*Green Perspective of Nano-Biotechnology: Nanotoxicity Horizon to Biomedical Applications,*” contributing to sustainable advancements in the field of nanotechnology.

